# Silico-tuberculosis, silicosis and other respiratory morbidities among sandstone mine workers in Rajasthan- a cross-sectional study

**DOI:** 10.1371/journal.pone.0230574

**Published:** 2020-04-16

**Authors:** Saranya Rajavel, Pankaja Raghav, Manoj Kumar Gupta, Venkiteswaran Muralidhar

**Affiliations:** 1 Department of Community Medicine and Family Medicine, All India Institute of Medical Sciences (AIIMS) Jodhpur, Jodhpur, Rajasthan, India; 2 Department of General Surgery, Chettinad Medical College and Hospital, Chennai, Tamil Nadu, India; KAOHSIUNG MEDICAL UNIVERSITY, TAIWAN

## Abstract

**Background:**

Exposures to respirable crystalline silica causes silicosis, pulmonary tuberculosis, chronic obstructive pulmonary disease, lung cancer, autoimmune disorders and chronic renal disease. The aim of this study was to find out the prevalence of silico-tuberculosis, silicosis and other respiratory morbidities in sandstone mine workers in Jodhpur district of Rajasthan.

**Methods:**

It was a cross-sectional study done in sandstone mines in Jodhpur. A total of 15 mines were selected. The sample size was calculated and fixed to 174 mine workers. Chi-square and t-test were applied to draw inferences.

**Results:**

The mean age of the mine workers was 39.13 ± 11.09 years. Three fourth (75.3%) of the workers were working for more than ten years in mines. Around 30.0% had a history of tuberculosis. Abnormal spirometry was found in 89.2% of workers. Around 42% of mine workers were found with abnormal chest x-rays. Prevalence of silicosis was 37.3%, silico-tuberculosis was 7.4%, tuberculosis was 10.0%, and other respiratory diseases like emphysema and pleural effusion were diagnosed among 4.3% workers.

**Conclusion:**

Prevalence of silico-tuberculosis, silicosis and other respiratory morbidities are high among sandstone mine workers.

## Introduction

Silicosis is a progressive and disabling interstitial lung disease caused by inhalation of crystalline silica and is irreversible. This incurable disease affects millions of workers engaged in hazardous dusty occupations in many countries. [1] World Health Organisation (WHO) 2002 report stated that, globally 30,000 people death and 12,88,000 Disability Adjusted Life Year (DALYs) loss occur annually due to pneumoconiosis caused by airborne particulates. [2] Asia contributes to two-thirds of the global work-related mortality, followed by Africa at 11.8% and Europe at 11.7%. [3] With its potential to cause progressive physical disability, silicosis continues to be one of the most important occupational health illnesses in the world. The prevalence of silicosis in India ranges widely from 3.5% in the ordnance factory to 54.6% in the slate-pencil industry [4] and this variation in prevalence is due to different silica concentrations in work environment, duration of exposure and job demands. There are many occupations in which workers may be exposed to crystalline silica like; mining, quarrying, construction work, glass including fibreglass industry, agriculture work, industries producing metal products etc., [5] The freshly fractured silica (i.e. blasting, drilling) has a significant health effect than aged silica or geologically ancient clays (fractured through the natural process). [6]

The occupational diseases associated with inhalation of respirable crystalline silica are silicosis, pulmonary tuberculosis, chronic bronchitis, emphysema, lung cancer, mycobacterial, fungal, and bacterial lung infections. Besides that, extra-pulmonary tuberculosis, autoimmune diseases or immunologic disorders (scleroderma, systemic lupus erythematosus, rheumatoid arthritis, sarcoidosis), chronic renal disease, subclinical renal changes and cancers are some of the extra-pulmonary diseases which are also associated with inhalation of crystalline silica dust. [1,7]. Tuberculosis, which is the clinical complication of the silicosis(called silico-tuberculosis), is still a significant public health concern in low and middle-income countries. [8] Silica-exposed mine workers with or without silicosis are at increased risk for tuberculosis. The risk of a patient with silicosis developing tuberculosis is higher (2.8 to 39 times) than a healthy individual. [9]

After extensive literature search, it was noticed that there is lack of studies depicting the exact prevalence of silicosis and other respiratory morbidities among sandstone mine workers in India. With this background, this study was planned to find out the prevalence of silico-tuberculosis, silicosis and other respiratory morbidities in sandstone mine workers in Jodhpur district of Rajasthan.

## Material and methods

It was a cross-sectional study which was conducted from June 2017 to May 2019 among mine workers who were exposed to silica dust in sandstone mines in Jodhpur, Rajasthan. The range of silicosis prevalence among stone quarries worker is 10% - 22%. [10,11] Considering the lower level of prevalence (10%) and assuming ± 5% permissible level of error in the estimation of this prevalence with 95% Confidence Interval, the sample size was calculated by Zα^2^ PQ/ L^2^. After adding 20% non-response rate, the final sample size came out was 174 mine workers.

**Fig 1:**
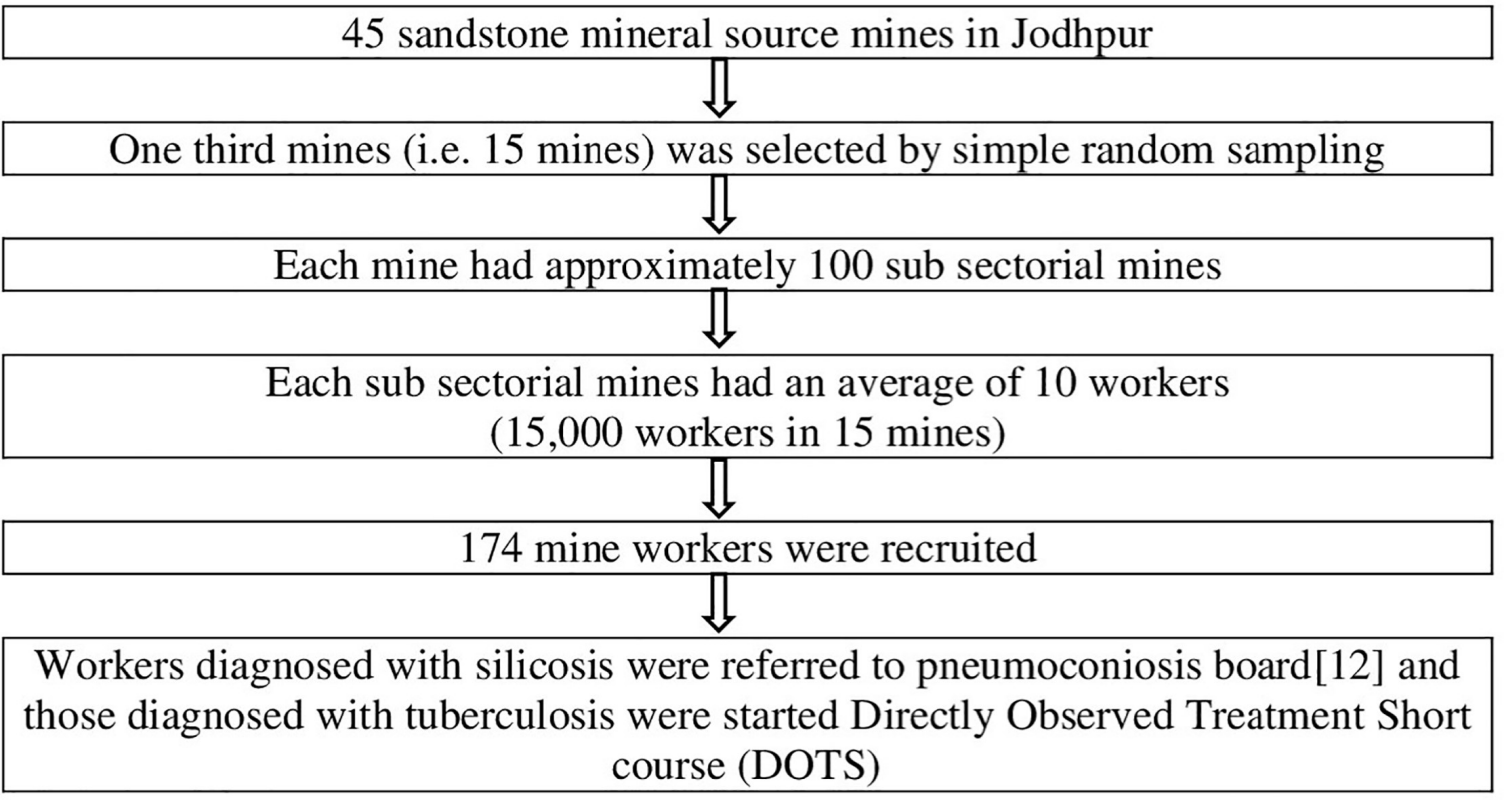
Flowdiagram of selection of mines, mine workers.

Fig 1 shows the process of selection of mines and mine workers. Out of 45 sandstone mines, 15 mines were selected by simple random sampling. The list of mine workers employed in each mine source was not available as the mine workers tend to shift their place of work frequently from one mine to another mine. Those mine workers who were available and willing to participate in the study were recruited until the sample size was achieved.

Ethical clearance was obtained from Institutional Ethics Committee, All India Institute of Medical Sciences (AIIMS) Jodhpur. Written informed consent was taken from all the mine workers who participated in the study. Assent was obtained from mine workers who were below 18-year of age and consent was taken from their parents.

All the selected mine workers were interviewed using pre-tested questionnaire and the following symptoms were recorded for suspected tuberculosis such as cough > 2 weeks, loss of weight, loss of appetite, evening rise of temperature, night sweats, blood in the sputum. Systematic examination of the respiratory system was also done to find any features of tuberculosis.

**Fig 2:**
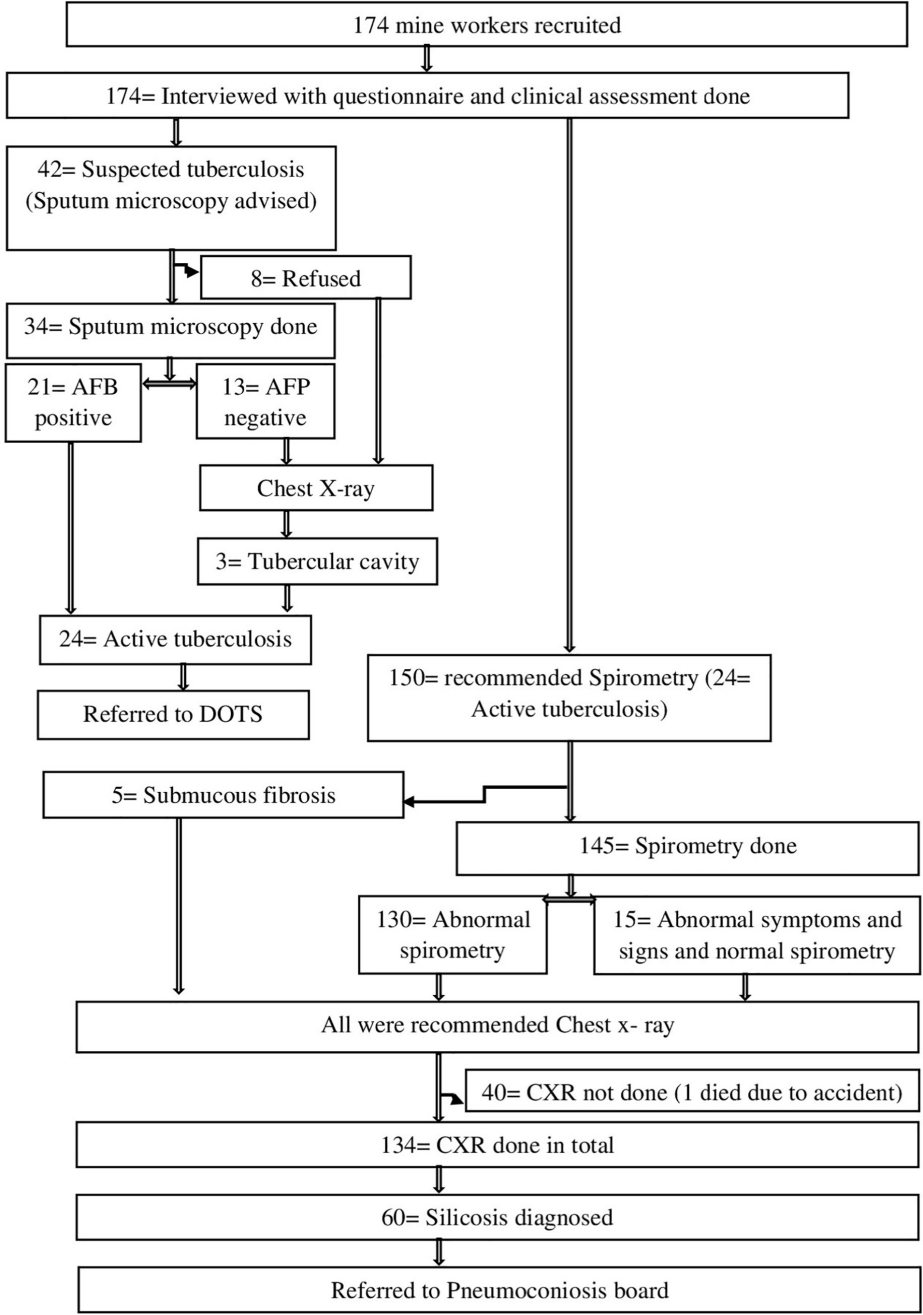
Flow diagram for investigation of mine workers.

To confirm the diagnosis of tuberculosis, sputum microscopy was done for the workers who had symptoms and signs suggestive of tuberculosis in nearby health centre. To assess the lung function, spirometry was done for all study participants, excluding those who had active tuberculosis. Spirometry of these participants were carried out using computerized spirometer. Forced expiratory volume in 1 second (FEV1) and Forced vital capacity (FVC) were recorded. Participants with FEV1/FVC < 70% and/or FVC predicted % < 80% were considered to have an abnormal spirometry. Participants who had a clinical symptoms and signs or abnormal spirometry were advised chest x-ray to diagnose silicosis and other respiratory disease as shown in Fig 2. Diagnosis of silicosis and silico-tuberculosis was made as per International Labour Organization (ILO) guidelines, 2011. Those with positive sputum smear were referred to district tuberculosis centre and with silicosis and silico-tuberculosis were referred to Pneumoconiosis board [12]. Data analysis was done using Statistical Package for Social Science (SPSS) software version 23. All the variables were analysed using descriptive statistics to calculate frequencies, mean, range etc. By considering 95% level of significance, chi-square test, Fischer exact test and t test were applied to determine the association between different variables. Median values were given for the variables with outliers. There were no missing data in the dataset.

## Results

The mean age of mine workers was 39.13 ± 11.09 years with three workers aged ≤, 18 years and nine workers were elderly. Out of 174 participants, 128 (73.6%) were males and 46 (26.4%) were females. Majority (90.8%) of them were married and almost half (49.4%) of the workers were illiterate and (82.2%) of the workers were from lower and lower middle socio-economic class as depicted in Table 1.

**Table 1:** Distribution of mine workers based on socio-demographic profile (N = 174).

Variables	N	%
**Age (years)**		
< 18	3	1.7
19–59	162	93.1
> 60	9	5.2
**Gender**		
Male	128	73.6
Female	46	26.4
**Marital status**		
Unmarried	10	5.7
Married	158	90.8
Widow	6	3.4
**Religion**		
Hindu	168	96.6
Muslim	6	3.4
**Caste**		
SC	158	90.8
OBC	16	9.2
**Type of family**		
Joint	89	51.1
Nuclear	85	48.9
**Socioeconomic status- Modified B.G. Prasad scale, 2018**		
І (Upper)	1	0.6
ІІ (Upper middle)	5	2.9
ІІІ (Middle)	25	14.4
ІV (Lower middle)	99	56.9
V (Lower)	44	25.3
**Education**		
Illiterate	86	49.4
Just literate/ below primary	18	10.3
Primary class	31	17.8
Middle class	23	13.2
Secondary class	10	5.7
Higher secondary and above	6	3.4

As shown in Table 2, the mean (SD) age of entry into mine was 20.25 (6.13) years and mean period of work by mine workers was 18.88 (9.81) years, ranged from 1.5 to 50 years. Male mine workers had worked for a mean (SD) year of 19.55 (9.98) (Range: 1.5–50), while female workers had worked for mean (SD) years of 17.02 (9.16) (Range: 2–36). Out of 174 study participants, 167 (96%) had worked for ≤ 8 hours per day in mine. Mine workers had worked for a mean (SD) hour of 7.09 (1.42) per day ranged from 2 to 12 hours

**Table 2:** Gender wise distribution of age of entry into mine and total year of work by mine workers in mines.

Variables	Male (%)	Female (%)	Total (%)	p-value
**Mean (SD) age of entry into mine**	19.54 (5.82)	22.23 (6.69)	20.25 (6.13)	0.01*
**Year of work**				
≤ 5	9 (7.0)	4 (8.7)	13 (7.5)	0.568^@^
6 to 10	20 (15.6)	10 (21.7)	30 (17.2)	
>10	99 (96.4)	32 (69.6)	131 (75.3)	
**Total**	**128**	**46**	**174**	

*Independent t-test value: 0.417 (df = 172)

^**@**^Chi-square value: 1.131 (df = 2)

Table 3 shows that majority (94.5%) of male workers were engaged in stone cutting, drilling or both, while most of the female workers (93.4%) were involved in loading stones, cleaning stone waste or both. Two participants were mine inspectors.

**Table 3:** Gender wise distribution of type of work done by mine workers.

Work Classifications	Type of work	Males (%)	Females (%)	Total (%)
High dust producing work	Stone cutting	26 (20.3)	3 (6.5)	29 (16.7)
	Stone drilling	25 (19.5)	0 (0)	25 (14.4)
	Stone cutting and drilling	70 (54.7)	0 (0)	70 (40.2)
Low dust producing work	Driver in mines	1 (0.8)	0 (0)	1 (0.6)
	Loading stones	1 (0.8)	11 (23.9)	12 (6.9)
	Cleaning stone waste	2 (1.6)	22 (47.8)	24 (13.8)
	Loading and cleaning stone waste	1 (0.8)	10 (21.7)	11 (6.3)
	Mine inspector	2 (1.6)	0 (0)	2 (1.1)
**Total**		**128**	**46**	**174**

Eight workers were already diagnosed with silicosis and 47 (27.0%) had tuberculosis for a median duration of 6 months. Workers with silicosis had worked in mines for mean (SD) period of 26.37 (7.42) years, ranged from 15 to 35 years as shown in Table 4.

**Table 4:** Distribution of mine workers according to their past respiratory morbidity profile (n = 174).

Variable		Duration of illness (years)			Treatment
	n (%)	Mean (SD)	Median	Range	n (%)
**Silicosis**	8 (4.6)	1.79 (1.55)	1.25	0.33 to 5	8 (100)
**Tuberculosis**	47 (27.0)	0.84 (1.41)	0.5	0.08 to 10	47 (100)
**Asthma**	4 (2.3)	2.39 (4.40)	0.24	0.08 to 9	3 (75.0)

On further analysis it was noted that, among 47 mine workers who received anti-tubercular therapy (ATT) once had a mean (SD) treatment duration of 6.78 (2.63) months, in which 17 (36.2%) received Category II treatment. The reasons noted for category II treatment were treatment failure and relapse. [13] The majority (76.6%) of the patients received treatment from government hospitals. As the number of ATT therapy increased, the mean duration of treatment as well as Category II treatment increased and there is a shift of health seeking behaviour towards government hospital than private hospital.

Out of 174 mine workers, 42 (24.1%) had symptoms suggestive of tuberculosis. Out of them, 34 had undergone sputum microscopy in which 21(61.8%) had sputum smear positive for Acid fast bacilli. Spirometry was done for 145 workers (24 workers diagnosed with active tuberculosis were excluded and five workers had difficulty in opening mouth because of submucosal fibrosis). Spirometry showed restrictive pattern in 113(78.0%) workers with the mean (SD) predicted FVC (%) of 62.42 (10.76) and it ranges from 28.2 to 98.4, followed by 17 (11.7%) had both obstructive and restrictive pattern. The mean (SD) value of FEV1/FVC (%) for the participants with obstructive pattern in spirometry was 63.42 (7.39), which ranges from 46.3 to 95.6. Out of 174 mine workers, 168 (96.5%) who had symptoms suggestive of silicosis or tuberculosis. Out of 168 workers, 134 (79.8%) could undergo chest x-ray and out of them, 74 (56%) had an abnormal x-ray findings. As shown in Table 5, the abnormalities in x-rays were significantly more among male workers (67.7%) than female workers.

**Table 5:** Distribution of mine workers based on various investigations.

Investigations	Males n (%)	Females n (%)	Total (%)	p value
Positive sputum smear **(n = 34)**	19 (61.3)	2 (66.7)	21 (61.8)	1.00^#^
Spirometry **(n = 145)**				
Normal	11 (10.7)	4 (9.5)	15 (10.3)	-
Restrictive pattern	78 (75.7)	35 (83.3)	113 (78.0)	
Both restrictive and obstructive pattern	14 (13.6)	3 (4.9)	17 (11.7)	
Abnormal Chest X-Ray **(n = 134)**	67 (67.7)	7 (22.0)	74 (55.2)	0.0001^@^

# Fischer exact value: -0.180 (df = 1)

^@^Chi-square value: 21.070 (df = 1)

**Fig 3:**
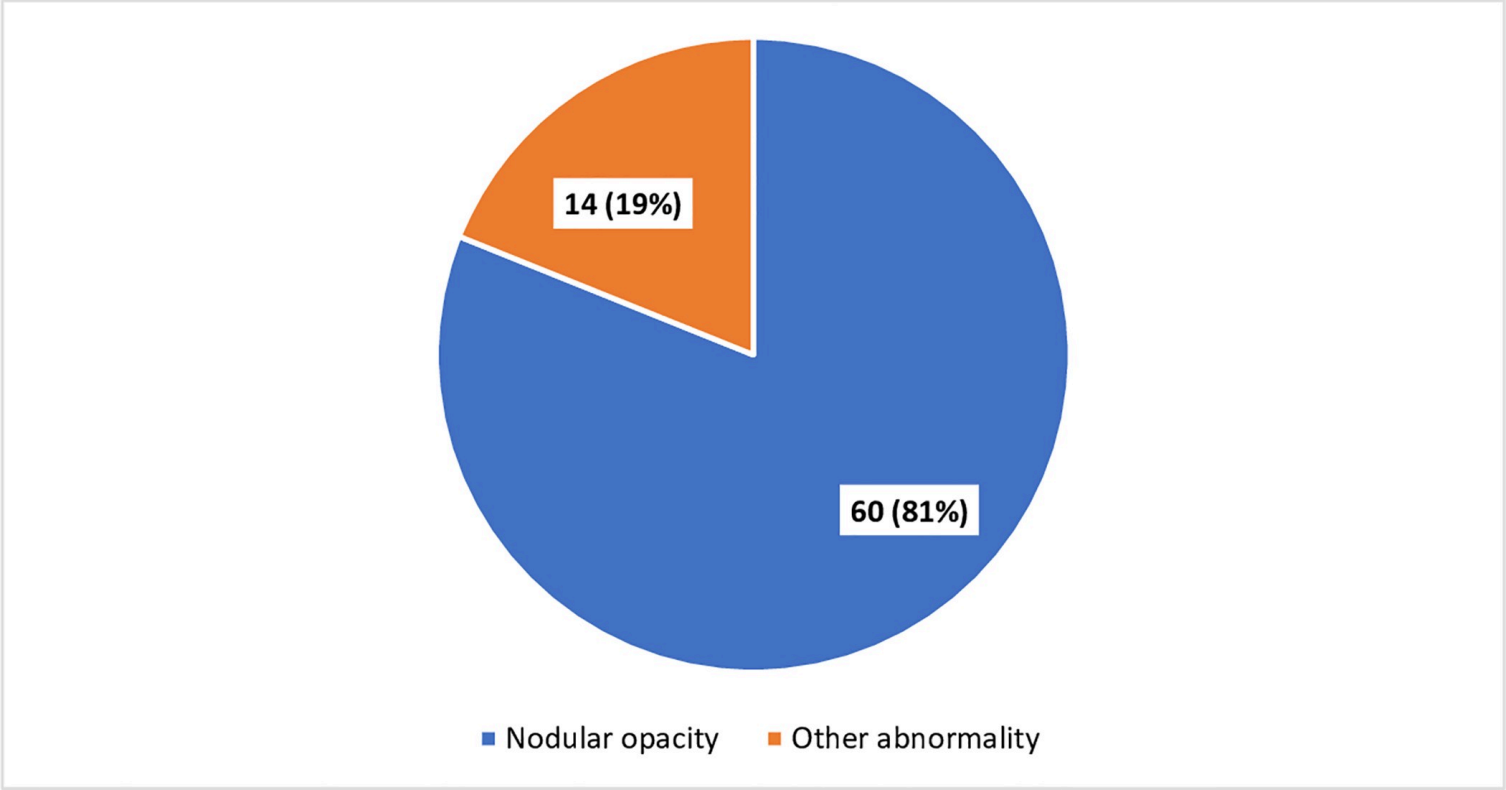
Abnormalities in chest Radiograph of mine workers (n = 74).

Fig 3 depicts, out of 74 mine workers who had abnormal x-ray findings, 60 (81%) had a reticulo-nodular opacity suggestive of silicosis while 14 (19%) had other abnormalities such as tuberculosis, emphysema, bronchiectasis, fibrosis of lung, lung collapse and pleural effusion.

**Fig 4:**
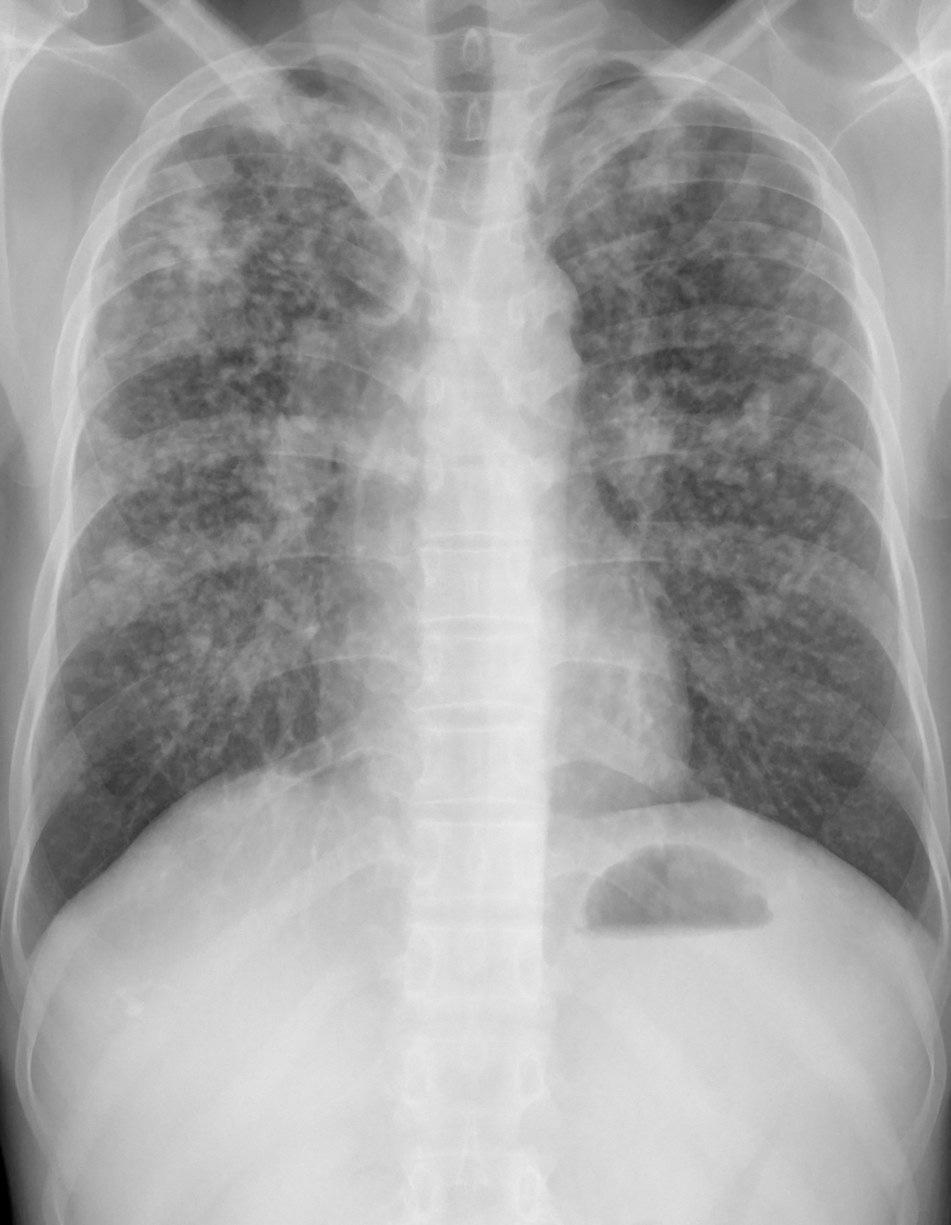
Chest X-ray of an sandstone mine worker.

Fig 4 shows the chest x-ray of an 40 year old sanstone mine worker who has worked for 18 years involved in stone cutting and drilling activities. The chest x-ray is an posteroanterior film with full inspiration and normal penetration. The trachea is in midline and both lungs shows the reticulo-nodular opacities in all the three zones. According to ILO classifications, they are primarily regular small opacities (q) with profusion of 3/+ and there was no involvement of pleura.

Table 6 illustrates that, out of 60 workers who had small nodular opacity, 12 (20%) had progressive massive fibrosis along with smaller opacities, in which 15% had large opacity longest dimension up to about 50 mm, 2% had large opacity having the longest dimension exceeding 50 mm but not exceeding the equivalent area of the right upper zone and 3% had large opacity exceeding the equivalent area of the right upper zone. Out of 60 workers who had smaller nodular opacity, majority had regular opacities (<1.5 to 10 mm). Most of the (86.6%) nodular opacities were seen bilaterally. Most of the chest x-ray showed involvement of all three zones, followed by two zone and single zone.

**Table 6:** Distribution of small nodular opacities in chest radiograph (n = 60).

Size of opacity	Primary small opacity		Secondary small opacity	
	n	%	n	%
p	35	58.3	31	51.7
q	14	23.3	16	26.7
r	6	10	7	11.7
t	3	5	1	1.7
u	2	3.3	5	8.3
**Total**	**60**	**100**	**60**	**100**
**Zone of involvement**	**Right side**		**Left side**	
	**n**	**%**	**n**	**%**
One zone	10	17.5	9	16.4
Two zones	13	22.8	16	29.1
Three zones	34	59.6	30	54.5
**Total**	**57**	**100**	**55**	**100**

**Fig 5:**
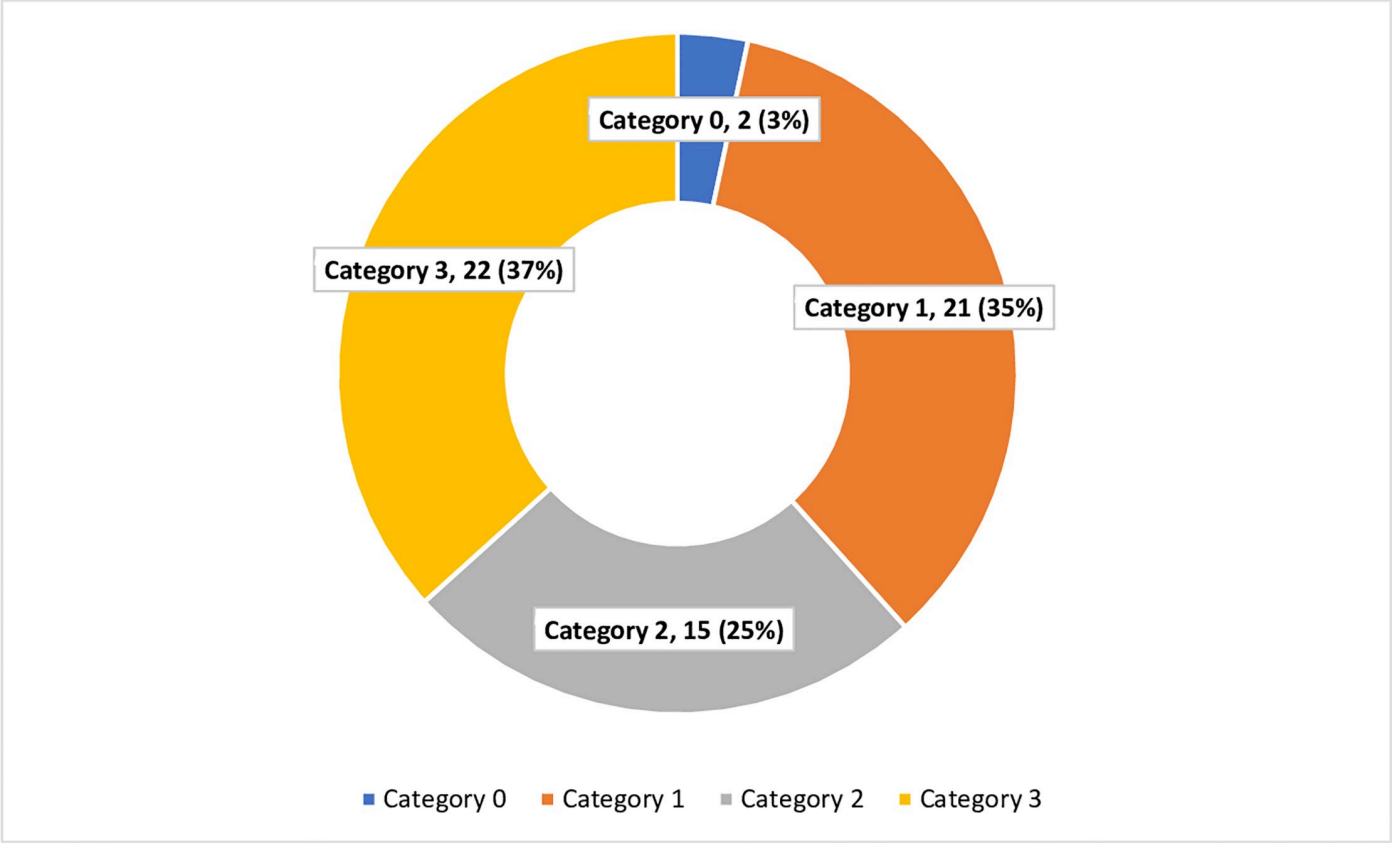
Profusion distribution of small nodular opacity in chest radiograph (n = 60).

Fig 5 depicts that, out of 60 radiographs which had nodular opacities, 22 (37%) had category 3 profusion, followed by category 2 and 1 (35% each) and 2 (3%) had category 0 profusion.

Table 7 illustrates the abnormalities detected in chest x-ray were hilar lymphadenopathy (12.7%), tuberculosis (11.2%), calcified hilar lymphadenpathy (8.2%), fibrosis of lung (6.7%) and emphysema (3.7%). Lung collapse, bronchiectasis and plueral effusion each was also observed in one x-ray.

**Table 7:** Various suspected findings in chest radiograph (n = 134).

Findings*	n	%
Normal	60	44.8 (36.6–53.2)
Silicosis	60	44.8 (36.6–53.2)
Tuberculosis	15	11.2 (6.9–17.6)
Hilar lymphadenopathy	17	12.7 (8.0–19.3)
Calcified hilar lymphadenopathy	11	8.2 (4.6–14.1)
Fibrosis of lung	9	6.7 (3.5–12.2)
Emphysema	5	3.7 (1.6–8.4)
Lung collapse	1	0.7 (0.1–4.1)
Bronchiectasis	1	0.7 (0.1–4.1)
Pleural effusion	1	0.7 (0.1–4.1)

*Multiple responses possible

Prevalence of silico-tuberculosis, silicosis is given for 134 workers who had undergone chest x-rays irrespective of sputum examination whereas prevalence for tuberculosis and other respiratory morbidity is given for 140 workers who had undergone chest x-rays (n = 134) and sputum examination without chest x-ray (n = 6). Table 8 illustrates the prevalence of silicosis in sandstone mine workers was 37.3%, silico-tuberculosis was 7.4%, tuberculosis was 10.0%, and other respiratory diseases 4.3% like emphysema and pleural effusion. Among workers with silicosis, one had silicosis with lung collapse, mostly probable cause of lung collapse would be spontaneous pneumothorax and among workers with silico-tuberculosis, one had associated bronchiectasis.

**Table 8:** Prevalence of Silico-tuberculosis, silicosis and other respiratory morbidities among mine workers.

**Prevalence**	**Male (n = 104) %**	**Female (n = 36) %**	**Total (n = 140)%**	**Chi-square value**	**p-value**
Normal	32 (30.8)	28 (77.8)	60 (42.9)	24.13	**0.0001**
Tuberculosis and other disease	17 (16.3)	3 (8.3)	20 (14.3)	1.40	0.283
**Prevalence**	**Male (n = 99) %**	**Female (n = 35) %**	**Total (n = 134) %**		**p-value**
Silicosis	46 (46.5)	4 (11.4)	50 (37.3)	13.57	**0.0001**
Silico-tuberculosis	9 (9.1)	1 (2.9)	10 (7.5)	1.45	0.453

## Discussion

Silicosis remains a worldwide public health problem as there is no proven treatment available, and the legislative action on preventive measures are lacking. Besides that, there is a lack of conclusive data to establish the true burden of silicosis. It thus becomes important for researchers to know the extent of burden of occupational silicosis. This study has brought out this information in the Jodhpur district of Rajasthan, India.

In the present study, almost half of the mine workers were belonging to 30 to 44 years age group with mean age of 39.13 ± 11.09 years. This finding is very much similar to the findings of the studies conducted by Ahmad A [14–16] in Rajasthan where author has analysed secondary data of 507 sandstone mine worker from National Institute for Miner's Health, Ministry of Mines.

In this study, near about three fourth of the mine workers were male. This gender distribution of mine workers is very much similar to the study conducted by Tiwari RR in Gujarat [17], but not by another study conducted by same author among quartz stone workers in which 55.1% were males and 44.9% were females. [18] Shamim M et al. also found the preponderance of male workers in sandstone mine workers in six districts of Rajasthan. [6]

Most of the mine workers belonged to the Scheduled caste category in the present study. This caste distribution is in accordance with the studies conducted by Ahmad A and Shamim M et al. [6]

The average family size of the mine workers was 6.63 ± 2.29 in the present study, which is almost similar to the studies conducted by Ahmad A. [14,15] This average family size is higher than the national average reported by National Family Heath Survey ІV. [19]

Nearly half of the mine workers were illiterate in the present study. A higher level of illiteracy among mine workers has also been reported by Tiwari RR et al. in Gujarat [17], Yadav S.P et al. [14] and Ahmad A in Rajasthan. [20] The majority (82.2%) of the workers belonged to lower and lower middle socio-economic class according to modified B.G. Prasad’s classification. Average monthly income of mine workers in the present study was Rs. 8929/- This was high compared to the study done by Ahmad A [14] among sandstone miners in which the average monthly income was Rs. 3200/-

In the present study, near about three fourth (75.3%) of the workers were working in mines for more than ten years. Similar kind of work experience by mine workers has been documented by Ahmad A. The mean years of working in the present study was 18.88 ± 9.81 which is lower than the mean years of work reported by Ahmad A [21] and Shamim M et.al. [6] The mean age of entry into mines for male worker was (19.54 ± 5.82) significantly lower than female worker (22.23 ± 6.69). This higher mean age of entrant into mine for female may be due to the reason that female start working in mines only after marriage or when their husband got diseased or died due to silicosis. The mean age of entrant for male may be lower because generation wise these families are following the patriarchal culture of working in mines. So, the male members start working at early age to earn for their family, giving very less priority to education.

In the present study, mine workers were working for a mean duration of 7.09 ± 1.42 hours per day which is in accordance with OSHA (Occupational Safety and Health Standards) and the Factory Act recommendations. Dixit R et al. [22] had reported the duration of work as six hours per day in a stone crushing factory in Rajasthan.

Male mine workers were mostly involved in high dust producing work in mines like stone cutting and drilling while female mine workers were mostly involved in low dust producing activities such as loading stones and cleaning stone waste. Stone cutting and drilling as the most common work pattern by miners has also been observed by Ahmad A [15] among sandstone mine workers in Rajasthan.

More than half of the mine workers (63%) had reported clinical symptoms in the present study, in which breathlessness was the most common and most frequent symptom. Athavale et al. in flour mill workers [23], Tyagi et al. in mine workers [24] and J Whig et al. [25] in a emery-polish workers have also reported breathlessness as most common symptom among workers. Other symptoms reported by mine workers were chest pain, dry cough, productive cough, haemoptysis, palpitations, easy fatiguability, evening fever and weight loss. A similar pattern of symptoms was observed in the studies conducted by Ahmad A [14,15], Tyagi et al. [24] and Tiwari RR et al. [17]

In the present study, 28.7% workers were underweight. This level of undernourishment among mine workers is lower than the level (38%) reported by Ahmad A. [15] In a study conducted by Chaudhury et al. [26] among 53 symptomatic workers, more than half of them were underweight, which is higher as compared to present study and the difference may be because of smaller sample size and inclusion of only symptomatic workers.

On clinical examination, almost one third of the workers had signs of anemia and twenty-five out of 174 were found hypertensive. Three fourth of the workers were having tachypnea (respiratory rate > 16 breathe per minute) which was significantly more among male workers. The majority (95.4%) of the mine workers were not able to expand their chest for > 5 cm on clinical examination. Nearly one fourth of the workers (26.4%) had some abnormal sounds on chest auscultation. Tyagi P et al. [24] has also observed the same among 36.2% of mine workers.

In the present study, 27.0% of mine workers had history of tuberculosis and eight were having history of silicosis. Workers with silicosis had worked in mines for an average period of 26.37 (7.42) years, ranged from 15 to 35 years.

Based on sputum microscopy and chest x-ray examination, 24 mine workers were found to have active tuberculosis. Almost 90% of the mine workers were having abnormal spirometry findings which was insignificantly different between males and females. This finding is similar to the findings of the study conducted by Sivanmani et al. [27] Tiwari RR et al. had reported less proportion of workers with abnormal spirometry as compared to present study. [17,28]

More than half of the mine workers in the present study were found to have abnormalities in chest x-ray, which was significantly more among male workers as compared to female workers. The most common abnormality detected was reticulo-nodular opacities with bilateral involvement of all three zones. Similar kinds of observation through chest x-rays have been made by Sivanmani et al. [27] in a stone crushing industry in Tamilnadu.

Prevalence of only silicosis in sandstone mine workers in the present study was 37.3%. This prevalence is quite higher than the prevalence of silicosis reported by Tiwari RR et al. [28] among female stone workers (14%), Shamim M et al. [6] among sandstone mine workers in Rajasthan (12%), Tiwar RR et al. [17] in ex-quartz crushing workers (17.9%), Singh SK [29] in sandstone workers of Rajasthan (22.2%) and Mathur ML [30] in Rajasthan(12.4%). Almost a similar level of prevalence of silicosis (38%) has been documented by National Institute of Miner’s Health for mine workers in Rajasthan through a collection of primary data. [31] Secondary data analysis by the same institute in the same year (2014) has reported a quite higher prevalence of silicosis (72%). [32]

In the present study, prevalence of silico-tuberculosis was 7.4%, which is quite lower than the prevalence reported by Shamim M et al. [6] and Tiwar RR et al. [17] Prevalence of only tuberculosis in the present study was 10.0% and this finding is comparable to the studies conducted by Tiwar RR et al. [17], Sivanmani et al. [27] and Tiwari RR et al. [33] Other respiratory diseases (4.3%) found in present study includes emphysema, pleural effusion, bronchiectasis and lung collapse. The most probable cause of lung collapse would have been spontaneous pneumothorax. This finding is similar to the studies conducted by Sharma R.K et al. [34], Mishra et al. [35], Bairagya et al. [36], Srivastava et al. [37], Fotedar et al. [38] and Gupta K B et al. [39]

The limitation of the present study is that out of 42 workers with suspected tuberculosis, eight workers did not undergo sputum examination but all of them had chest x-ray. Out of eight workers, three had shown tubercular cavity for which treatment were initiated and counted for prevalence. Five workers did not show any tubercular findings in CXR. These eight workers were contacted thrice at home and repeated phone calls were also made. The non-compliance of the five workers (11.9%) for sputum smear examination, would have slightly affected the prevalence of tuberculosis in this study.

## Conclusion & recommendations

Respiratory morbidities like silicosis, silico-tuberculosis, tuberculosis, emphysema, pleural effusion and lung collapse were assessed among mine workers through clinical assessment, spirometry, sputum examination and chest x-rays. The prevalence of those respiratory morbidities among sandstone mine workers was very high (57.1%). Clinical examination and investigations like spirometry, chest x-ray and sputum microscopy should be done on a regular frequency for mine workers, in order to assess respiratory morbidities at an early stage.

## Supporting information

S1 Questionnaire (DOCX)
