# Supplementary material for: Silico-tuberculosis, silicosis and other respiratory morbidities among sandstone mine workers in Rajasthan- a cross-sectional study
Source: PLoS One. 2020 Apr 16;15(4):e0230574. doi: 10.1371/journal.pone.0230574 (PMC7162522; doi:10.1371/journal.pone.0230574)
Supplement: S1 Questionnaire — (DOCX) [file pone.0230574.s001.docx]

| **All India Institute of Medical Sciences Jodhpur, Rajasthan** Department Of Community Medicine And Family Medicine |
| --- |

Silico-tuberculosis, silicosis and other respiratory morbidities in mine workers

Serial Number: __________ Place: ___________ Date: __________ Mobile Number: ___________________

**Section A – Personal Information**

1.Name: Last name: Father name:

2. Age

3. Sex: Male/ Female/ Others

4. Address:

5. Urban/ Rural:

6. Village/ Ward name :

7. Residence of village since:

8. Marital status: Unmarried/ Married/ Widower/Separated

9. Education:

10. Religion:

11. Caste:

12. Type of family: Nuclear/ Joint

13. Total Family members:

14. Total income of the family per month:

**Section B – Occupational Information**

15. Present Occupation:

16. Employee/ Badge number:

17. Designation in mine:

18. Employer Name:

19. Mine Address:

20. Details of occupation:

S.No

Years

(From-To)

Type of

Occupation

Work

hours/day

Main

task

done

Have

you

faced

any

injuries

Description

of injury (if

any)

Total

duration

**Section C**

21. After work, are you showering (Y/N):____

22. After work, are you changing clothes (Y/N):____

23. How long it will take to reach worker’s house from mine:_____ (Distance in m/Km)

24. In case of female worker, whether mother is accompanied with child during working hours?: Yes / No

1. If yes, what is the age of child:
2. How many years child has been exposed:
3. How many hours child is being exposed per day:

**Section D – Illness History**

25. Are you currently suffering from any respiratory problem (Y/N)______ If no then go to Question 29

26. Do you have any respiratory complaints from the list given below? (tick multiple options if needed)

| S. No | Symptoms | Yes/ No | If yes, Duration | Continuous/ Recurrent | If recurrent, frequency (in last 6 months) | Trend of symptoms  (Increasing/decreasing/no change) |
| --- | --- | --- | --- | --- | --- | --- |
| 1 | Breathlessness |  |  |  |  |  |
| 2 | Dry cough |  |  |  |  |  |
| 3 | Productive cough |  |  |  |  |  |
| 4 | Cough with blood discharge |  |  |  |  |  |
| 5 | Chest pain |  |  |  |  |  |
| 6 | Palpitation |  |  |  |  |  |
| 7 | Weakness |  |  |  |  |  |
| 8 | Any other |  |  |  |  |  |

27. Do you have any complaints from the list given below? (tick multiple options if needed)

| S. No | Symptoms | Yes/ No | If yes, Duration | Continuous/ Recurrent | If recurrent, fequency (in last 6 months) | Trend of symptoms  (Increasing/decreasing/no change) |
| --- | --- | --- | --- | --- | --- | --- |
| 1 | Evening fever |  |  |  |  |  |
| 2 | Weight loss |  |  |  |  |  |
| 3 | Any other |  |  |  |  |  |

28. Explain when do you feel breathless? Grade

1. After strenuous exercise / work 0
2. After doing moderate activity or walking up the slope or climbing two flight of stairs I
3. After walking on plain surface for 8-10 minutes (500 mtrs) II
4. After walking on plain surface for less then 500 m or while normal daily living activity III
5. Even at the rest IV

29. Is there history of any of the following diseases?

| S. No | Disease | Yes/ No | Duration | Treatment (Medication) | Present status (Complete/ Incomplete/ On treatment) |
| --- | --- | --- | --- | --- | --- |
| 1 | Silicosis |  |  |  |  |
| 2 | Tuberculosis (with Frequency) |  |  |  |  |
| 3 | Silico-tuberculosis |  |  |  |  |
| 4 | Asthma |  |  |  |  |
| 5 | COPD |  |  |  |  |
| 6 | Chronic Bronchitis |  |  |  |  |
| 7 | Cancer |  |  |  |  |
| 8 | Diabetes |  |  |  |  |
| 9 | Hypertension |  |  |  |  |
| 10 | Any Surgery |  |  |  |  |
| 11 | Any Hospitalization |  |  |  |  |

30. Have you received Anti-tubercular treatment before? Yes / No

31. If yes, how many times you have been treated with Anti-tubercular treatment? ________

32.

| No. of treatment | For how long the treatment was taken | H/o injection (Yes/No) | From Where (Place of treatment) |
| --- | --- | --- | --- |
| 1 |  |  |  |
| 2 |  |  |  |
| 3 |  |  |  |

**Section E – Smoking, alcohol, opium History**

| 33. a. Have you ever smoked ( Y/N)_____  b. Are you currently smoking….Y/N  c. Which form of tobacco you use routinely (Smoke/smokeless) ___  d. How long have you smoked for (yrs)____  e. How long ago did you stop (Yrs)____  f. How many cigarettes or Beedies do you /used to smoke per day (Number)__________  34. a. Have you ever chewed tobacco/tobacco product (Y /N)  b. Are you currently chewing….. Y/N  c. How many times/ day __________  d. How many packets/ day ________ | e. How long have you used tobacco products(yrs)  f. How long ago did you stop (Yrs)____  35. a. Have you ever taken alcohol ( Y/N)_____  b. Are you currently taking alcohol (Y/N)_____  c. How long have you taking alcohol (yrs)____  d. How long ago did you stop (Yrs)____  e. How much do you /used to drink per day (in ml)__________  36. a.Have you ever taken opium ( Y/N)_____  b. Are you currently taking opium (Y/N)  c. How long are you taking opium (yrs)____  d. How long ago did you stop (Yrs)____  e. How much you will consume per day (in gm)__________ |
| --- | --- |

**Section F – Clinical Examination:**

37. General Physical Examination:

| a.Pallor (Y/N) | b. Icterus (Y/N) | c.Cyanosis (Y/N) | d. Clubbing (Y/N) | e.Lymphadenopathy (Y/N) | f. If yes, describe: |
| --- | --- | --- | --- | --- | --- |
| g. Height(cm) | h. Weight(Kg): | i. BMI | j.Waist circumference: | k.Hip circumference: | l. Waist hip ratio: |

38. Vital Signs:

a. Pulse:_________ b. Blood Pressure:________ c. Respiratory Rate:___

39. Systemic Examination: (Respiratory System)

| a. Inspection : Size | b. Shape | c. Symmetry of the chest: |  |  |
| --- | --- | --- | --- | --- |
| d. Palpation: Chest expansion: | e. Percussion | f. Auscultation: |  |  |
|  |  |  |  |  |
| **Section G: PFT performance Results:-** | | | | |

40. Spirometry:

|  | I | II | % of Pred. Value |
| --- | --- | --- | --- |
| FVC |  |  |  |
| FEV1 |  |  |  |
| % FEV1/FVC |  |  |  |
| PEFR |  |  |  |
| 25-75 |  |  |  |

**Section H– Dust Control Measures: (Observation)**

41. Wet drilling: Yes/No If yes, Water supply: Continuous / Intermittent

42. Dry drilling with dust collecting system: Yes/No If yes, Functional / Non-functional

43. Amount of dust present in the mine:

**Section I– Personal Protective Equipment: (Observation)**

| **S.No** | **Personal Protective Equipment** | **Yes/No** | **Using correctly/not** |
| --- | --- | --- | --- |
| 44 | Eye: Googles |  |  |
| 45 | Ear: Ear plugs |  |  |
| 46 | Face shield/ Mask |  |  |
|  | If yes, type of mask |  |  |
| 47 | Head: Hard hats |  |  |
| 48 | Coated fabric glove |  |  |
| 49 | Body protection: (like paper like fiber/ treated wool and cotton etc.) |  |  |
| 50 | Safety shoes |  |  |

**Section J – Investigation:**

51. SPo2 : Pre______ 52. SPO2 Post exercise:_______ 53. Blood Sugar:_______

54. Sputum microscopy: 55. Chest X Ray:

Diagnosis:___________________ Signature of investigator:____________
